# Methylated Septin 9 and Carcinoembryonic Antigen for Serological Diagnosis and Monitoring of Patients with Colorectal Cancer After Surgery

**DOI:** 10.1038/s41598-019-46876-4

**Published:** 2019-07-17

**Authors:** Zhi Yao Ma, Wai Lun Law, Enders Kai On Ng, Cherry Sze Yan Chan, Kam Shing Lau, Yuen Yee Cheng, Vivian Yvonne Shin, Ava Kwong, Wai K. Leung

**Affiliations:** 1 Department of Medicine, University of Hong Kong, Queen Mary Hospital, Hong Kong, China; 2 Department of Surgery, University of Hong Kong, Queen Mary Hospital, Hong Kong, China; 3 DiagCor Bioscience, Kowloon Bay, Hong Kong, China; 4 Asbestos Diseases Research Institute, Sydney Medical School, The University of Sydney, Sydney, Australia

**Keywords:** Gastroenterology, Colorectal cancer

## Abstract

With the increasing incidence and mortality of colorectal cancer (CRC), early and accurate diagnosis is of paramount priority to combat this cancer. Epigenetic alterations such as DNA methylation are innovative biomarkers for CRC, due to their stability, frequency, and accessibility in bodily fluids. In this study, blood samples were prospectively collected from patients before and after operation for CRC for determination of methylated septin 9 (mSEPT9) and compared to carcinoembryonic antigen (CEA). The sensitivity of using mSEPT9 methylation status for diagnosing CRC was significantly higher than using elevated CEA levels (73.2% vs 48.2%; p value < 0.001). The sensitivities of both tests increased with higher tumor staging (P = 0.004 and 0.04 respectively). Combined mSEPT9 and CEA had higher accuracy than single CEA or mSEPT9 (P = 0.009 and 0.532 separately). An increase in the methylation level of mSEPT9 detected in the post-operative samples was associated with a higher mortality rate (15.2% vs 1.8%; P = 0.024) and the presence of metastasis (27.3% vs 7.0%; P = 0.013). mSEPT9 was more sensitive than CEA for diagnosing CRC, and combined mSEPT9 and CEA was more accurate. After curative resection, detection of increased mSEPT9 methylation level may indicate adverse outcomes.

## Introduction

The incidence and mortality rates of colorectal cancer (CRC) has increased 10-fold globally, with an expected 60% increase by 2030^1^. In Hong Kong, the incidence of CRC has surpassed lung cancer and emerged as the most common cancer. The mortality of CRC also ranked second, with crude rates of 35 and 22.8 per 100,000 men and women, respectively^2^. Due to the rapidly rising incidence and mortality of CRC, early and accurate detecting methods are of high importance. Currently, colonoscopy is the most direct method for detecting colorectal neoplasm. However, many people may not accept colonoscopy due to its potential risk and discomfort. In comparison, non-invasive blood test appears to be simple and may have a higher compliance as a screening test, and will permit frequent monitoring after diagnosis and treatment as well. As described in a German study, 63.4% (109/172) of patients refused screening colonoscopy, whereas 82.6% (90/109) of them chose blood test instead^3^.

Aberrant methylation is a regulatory mechanism of gene expressions which is commonly found in tumor suppressor genes in many cancers, including CRC^4–6^. Various epigenetic biomarkers have been identified in previous studies for the diagnosis as well as predicting prognosis of CRC^7–9^. Among various methylated genes, methylated septin 9 (mSEPT9) is found to have extremely high methylation levels in colorectal tumor tissues^10^ and has been studied for serological diagnosis of CRC with high sensitivity^11–13^. Detection of circulating mSEPT9 DNA in blood has been recently approved by the Food and Drug Administration (FDA) of the United States. This assay provided an alternative non-invasive test for the screening of CRC and is commercially available^14^. However, the utility of mSEPT9 for monitoring CRC patients after surgery has not yet been determined and there are few researches comparing carcinoembryonic antigen (CEA), a well-established tumor marker for CRC, with mSEPT9.

CEA is an oncofetal antigen generated from endodermal epithelial tumor cells in the alimentary tract^15^. The American Society of Clinical Oncology recommends that CEA to be measured every 3 months for at least 3 years in stage II and III CRC patients post-operatively^16^. Data are however insufficient to recommend other blood-based tumor markers for the monitoring of CRC patients at this stage.

The current study is a prospective study that aims to (1) evaluate the diagnostic accuracy of detecting mSEPT9 DNA in the blood of patients with different stages of colorectal neoplasm as compared to CEA and (2) to determine the role of mSEPT9, when compare to that of CEA, in monitoring CRC patients who have undergone curative resection of tumor.

## Results

### Participants

**Figure 1:**
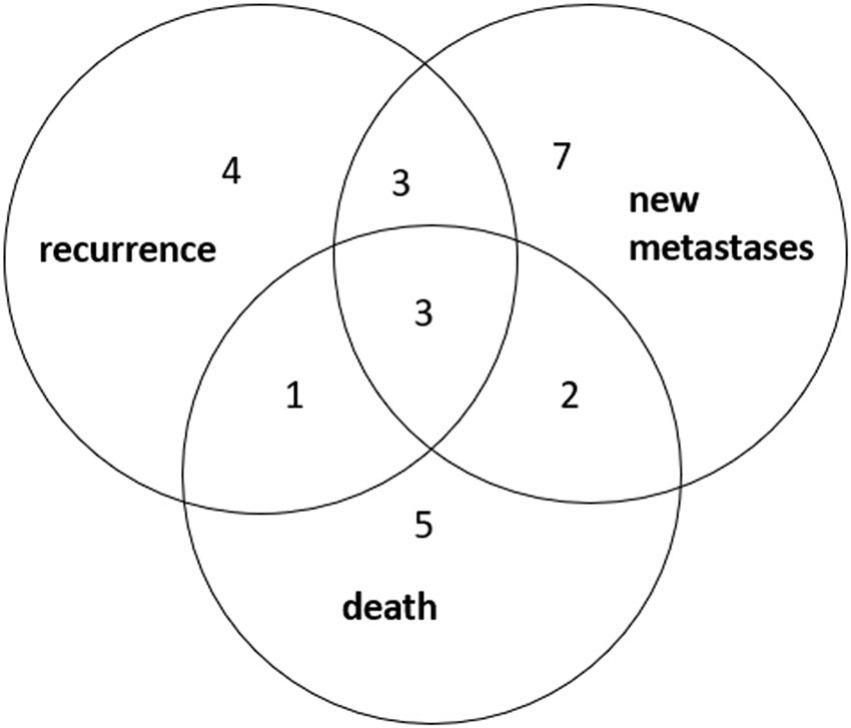
Number of cases with recurrence, new metastases and death during follow-up. Non-overlapped domain stands for colorectal cancer cases who had one poor prognostic outcome of recurrence, new metastases, and death; while the overlapped domain representing cases who had more than one.

A total of 282 patients were included in the first part of this study for determination of diagnostic values of mSEPT9, including 117 patients with confirmed CRC, 45 patients with advanced adenoma, 50 patients with non-advanced adenoma and 70 patients with normal colonoscopy (Table 1). The percentage of male patients was 62.8% and the mean age of patients was 66.1 (SD 11.5) years. Among the 117 confirmed CRC patients, 98 patients who had serial blood taken before and after surgical resection were included in the second part of this study for characterization of the role of mSEPT9 on post-operative monitoring (Table 2). During the follow-up of up to 28 months (mean 18.80 ± 5.87), we observed 11 cases with recurrence, 15 cases with new metastases and 11 cases with CRC associated death (Fig. 1).

**Table 1:** Patients’ characteristics.

	No.*	Mean age^#^ (SD)	Male (%)	No. of tested mSEPT9^**^	No. of tested CEA^**^
Colorectal Cancer	117	67.3 (12.8)	80 (68.4%)	112	110
Stage I	20	67.2 (10.1)	15 (75.0%)	19	19
Stage II	47	69.6 (12.8)	32 (68.1%)	46	45
Stage III	35	66.1 (13.4)	23 (65.7%)	33	32
Stage IV	4	69.8 (8.3)	3 (75.0%)	4	4
Unknown	11	60.6 (15.7)	7 (63.6%)	10	10
Colorectal Adenoma	95	66.8 (9.0)	58 (61.1%)	92	90
Advanced	45	67.3 (8.6)	31 (68.9%)	43	40
Non-advanced	50	66.4 (9.5)	27 (54.0%)	49	50
Normal	70	63.1 (11.7)	39 (55.7%)	69	58
Total	282	66.1 (11.5)	177 (62.8%)	273	258

^*^No. means the number of cases.

^#^Age was calculated at the time of diagnosing.

^**^No. means the number of cases which had results of mSEPT9 (methylated septin 9) or CEA (carcinoembryonic antigen), and the corresponding sensitivity was shown in Fig. 2.

**Table 2:** Characteristics of 98 colorectal cancer patients with serial follow up blood samples.

Stage	I	II	III	IV	Unknown	Total
Number	20	41	30	1	6	98
Male (%)	15 (75.0%)	28 (68.3%)	19 (63.3%)	0 (0.0%)	3 (50.0%)	65 (66.3%)
Mean Age (SD)	67.2 (10.1)	69.9 (13.1)	65.8 (13.7)	66.0	54.0 (16.9)	67.1 (13.3)
Location of tumor(s):						
Proximal^*^	4	12	4	0	0	20
Distal	16	28	26	1	6	77
Synchronous	0	1	0	0	0	1

^*^Proximal refers to lesion above the splenic flexure.

### Performance of mSEPT9 for diagnosis of colorectal neoplasm

**Figure 2:**
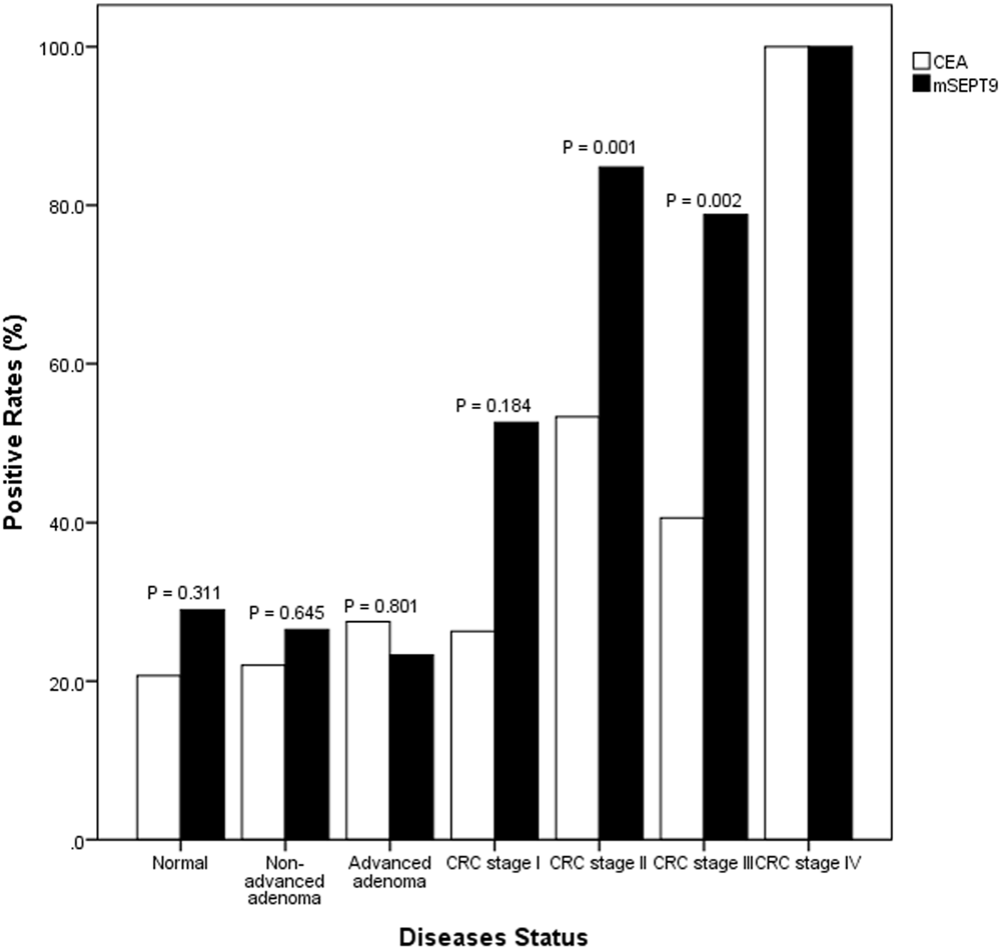
Positive rates of CEA and mSEPT9 in different patient groups. Sensitivities of carcinoembryonic antigen (CEA) and methylated septin 9 (mSEPT9) for detecting different stages of colorectal neoplasm; i.e. non-advanced adenoma (22.0% vs 26.5%; P = 0.645), advanced adenoma (27.5% vs 23.3%; P = 0.801), colorectal cancer (CRC): stage I (26.3% vs 52.6%; P = 0.184), stage II (53.3% vs 84.8%; P = 0.001), stage III (40.6% vs 78.8%; P = 0.002), and stage IV (100.0% vs 100.0%, p value is not available). In addition, the first pair of bars represented for false positive rates in participants with normal colonoscopy.

In the first part of this study, we determined the sensitivities of mSEPT9 and CEA for patients with different colonoscopic diagnoses and tumor stages. The overall positive rates of mSEPT9 and CEA in the pre-operative blood samples of patients with confirmed CRC were 73.2% (95% CI, 65.0–81.1%) and 48.2% (95% CI, 38.1–57.3%; P < 0.001), respectively. There was a progressive increase in the detection rate for both mSEPT9 (P = 0.004) and CEA (P = 0.04) with higher tumor staging (Fig. 2). The positive rate of mSEPT9 increased from 52.6% in patients with stage I cancer to 100% in patients with stage IV cancer whereas the corresponding positive rate of CEA increased from 26.3% to 100% in these patients. There was a significant difference in the positive rates between mSEPT9 and CEA in stage II (P = 0.001) and stage III (P = 0.002) cancers, but not in patients with stage I (P = 0.184) and IV cancers. However, the sensitivities of both mSEPT9 and CEA were low (<27.6%) in patients with adenoma, both advanced and non-advanced, and there was no significant difference in positive rates between mSEPT9 and CEA for patients with adenoma. The overall specificity of mSEPT9 and CEA in colonoscopy-negative subjects was comparable (71.0% and 79.3%; P = 0.311). Table 3 compared the accuracy of mSEPT9, CEA and combination of the two for patients and controls who had both test results available. The combined results, when either one positive test is treated as positive, showed a higher accuracy than CEA (P = 0.009) or mSEPT9 (P = 0.532) alone. Positive CEA was more commonly found in proximal than distal CRC (72.2% vs 39.2%; P = 0.017), while positive mSEPT9 showed no significant difference between proximal and distal cancer (90.0% vs 69.4%; P = 0.085).

**Table 3:** Accuracy of mSEPT9, CEA and the combination in detecting colorectal cancer.

Tests	Sensitivity	Specificity	Accuracy
mSEPT9	71.8% (77/105)	66.7% (38/57)	71.0% (115/162)
CEA	50.5% (53/105)	78.9% (45/57)	60.5% (98/162)
Combination^#^	85.7%	54.4%	74.7%
P value			
mSEPT9 vs CEA (2-sided)	0.001	0.206	0.061
mSEPT9 vs CEA (1-sided)	0.001	0.103	0.030
mSEPT9 vs Combination (2-sided)	0.039	0.250	0.532
mSEPT9 vs Combination (1-sided)	0.020	0.125	0.266
CEA vs Combination (2-sided)	0.000	0.009	0.009
CEA vs Combination (2-sided)	0.000	0.005	0.004

^#^Combination refers to either mSEPT9 (methylated septin 9) or CEA (carcinoembryonic antigen) was positive among participants who had both tests done (n = 162).

### Role of mSEPT9 and CEA in monitoring of cancer patients after surgery

**Figure 3:**
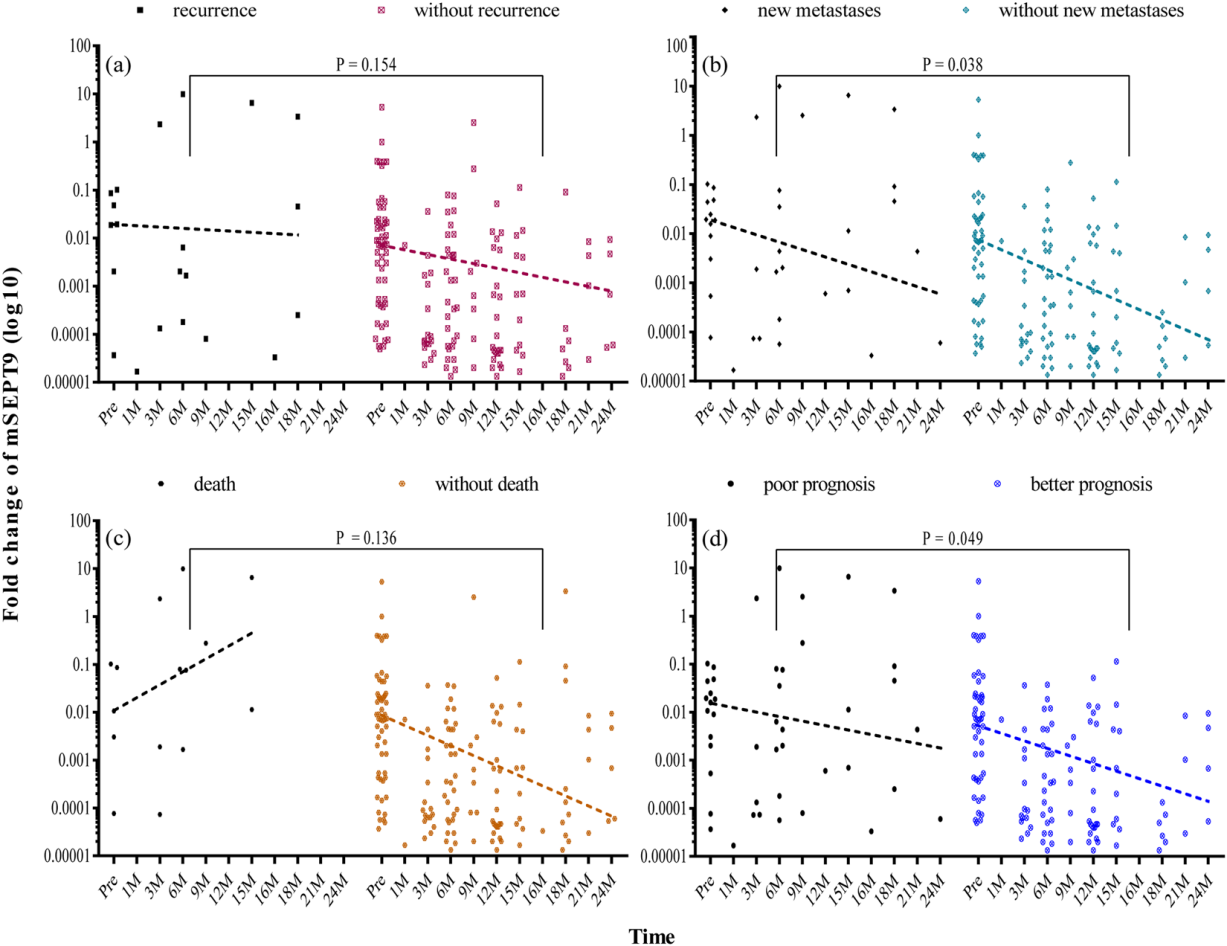
Relative methylation levels of mSEPT9 in colorectal cancer at different time points. Relative methylation of mSEPT9 (line = median) in colorectal cancer (CRC) patients (**a**) with or without recurrence; (**b**) with or without new metastases; (**c**) with or without death; (**d**) with poor or better prognosis during follow-up after operation. Poor prognosis refers to the presence of any of the three mentioned prognostic factors, while better prognosis refers to none of them. The P value was calculated by paired t test through GraphPad Prism (version 7; GraphPad Software, San Diego, CA) and represented the significance level of the difference in each group as aforesaid. The dots represent the mean value of SEPT9 methylation level in three PCR reactions for each individual patient, while the dotted lines show the trend of the median methylation levels with time. Pre = pre-operation, M = months.

**Figure 4:**
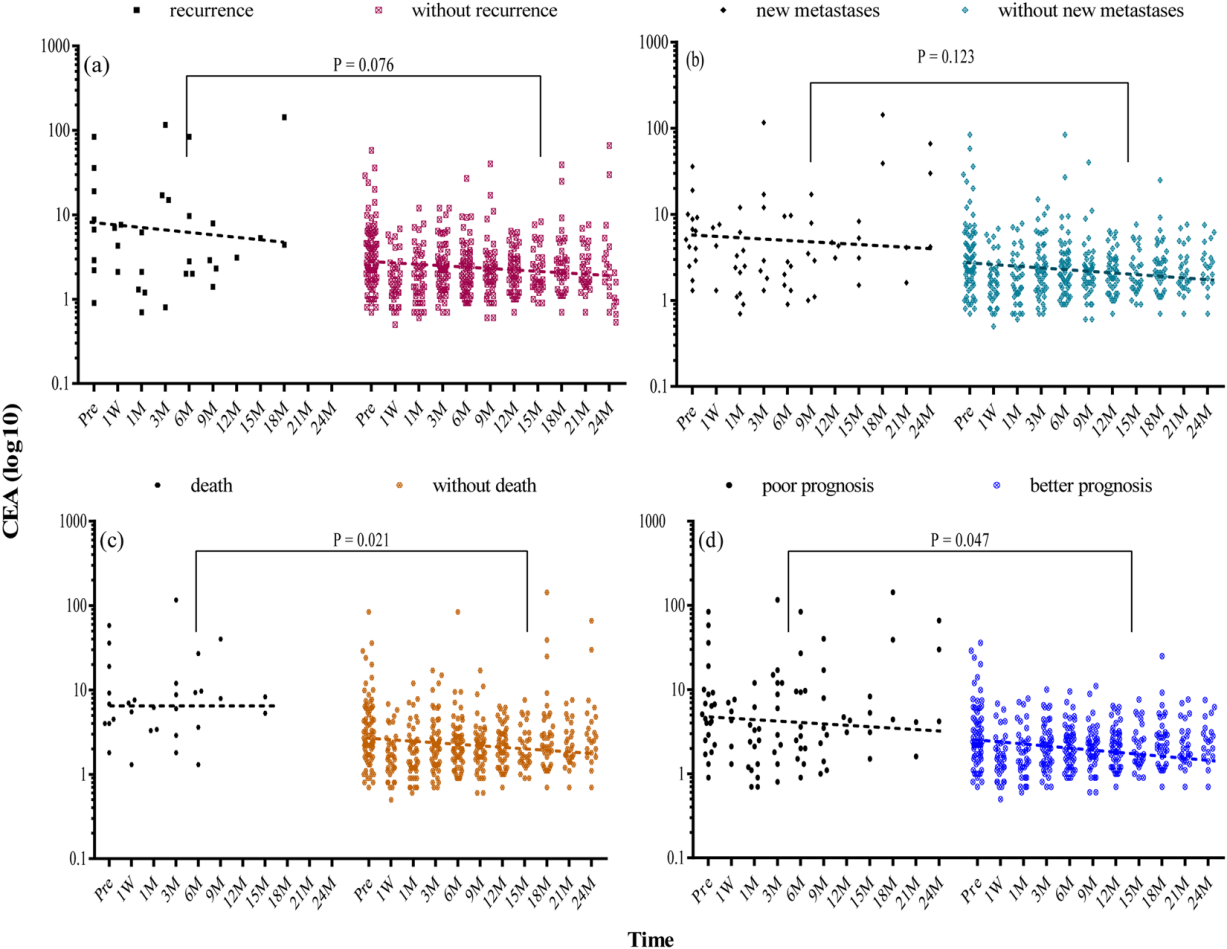
Distribution of carcinoembryonic antigen in colorectal cancer at different time points. Levels of carcinoembryonic antigen (CEA) (line = median) in colorectal cancer (CRC) patients (**a**) with or without recurrence; (**b**) with or without new metastases; (**c**) with or without death; (**d**) with poor or better prognosis during follow-up after operation. Poor prognosis refers to the presence of any of the three mentioned prognosis, while better prognosis refers to none of them. The P value was calculated by paired t test through GraphPad Prism (version 7; GraphPad Software, San Diego, CA) and represented the significance level of the difference in each group as aforesaid. The dots represent individual CEA value while the dotted lines show the trend of the median CEA levels with time. Pre = pre-operation, M = months.

**Figure 5:**
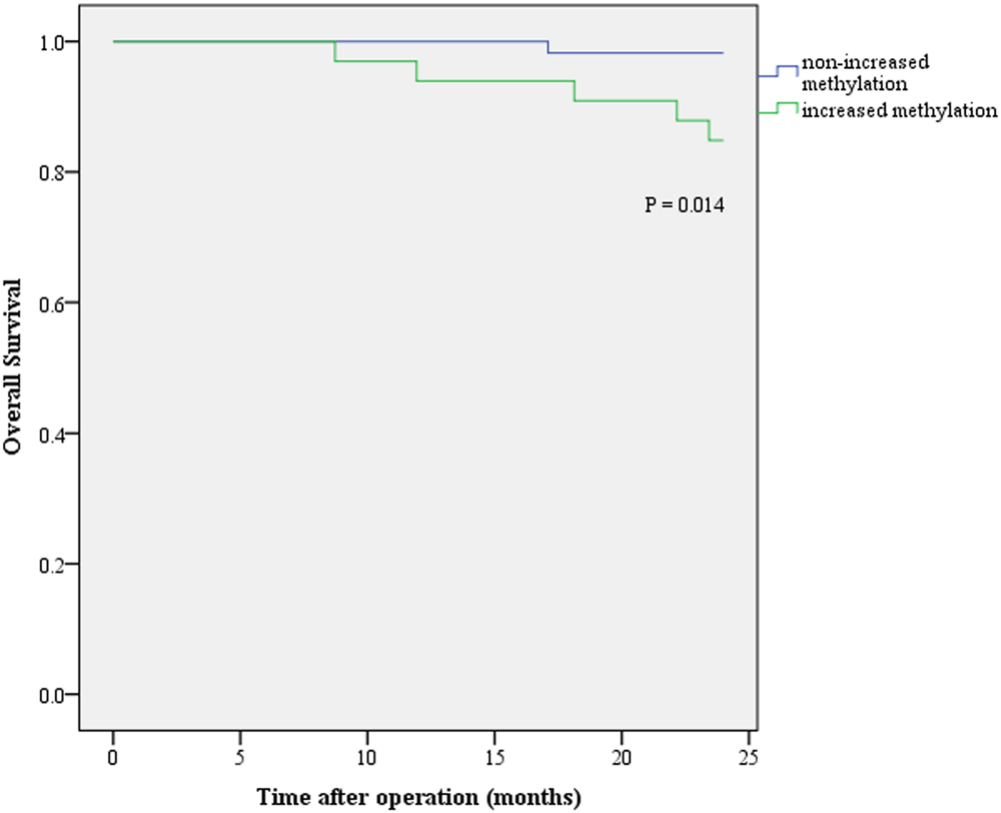
Overall survival in colorectal cancer patients after operation. The green and blue lines stand for the patient groups with and without increased methylation level after operation, respectively. Censor time is the death date or till the 24-month after surgery.

We also determined the changes in CEA and mSEPT9 in the post-operative blood samples of patients after resection of the tumor (Tables S1 amd S2). Overall, the proportion of patients with negative CEA was significantly higher than the proportion of patients with negative mSEPT9 at 6-months (71.8% vs 55.3%; P = 0.035) and 12-months (68.1% vs 48.1%; P = 0.028), especially for cases with non-advanced cancer (stage I/II) at 6-months (79.2% vs 55.6%; P = 0.013), and those without evidence of clinical recurrence at 6-months (72.9% vs 55.3%; P = 0.038) and 12-months (69.7% vs 49.0%; P = 0.033). This trend extends to 18-months and 24-months after operation although the difference did not reach statistical significance (Table 4). Among cancer patients with a positive mSEPT9 or CEA at baseline, 46.8% (mSEPT9) and 46.7% (CEA) turned negative at 6-months after operation (P = 1.0). These changes remain similar at 12-months, 18-months and 24-months (all P > 0.5). Figures 3 and 4 showed the distribution for different groups of mSEPT9 and CEA before and after operation. Patients with poor prognosis including recurrence, new metastases and death after operation had higher value of both mSEPT9 and CEA than those with better prognosis. When considering dynamic quantitative changes of mSEPT9 after operation, cases with increased methylation level had higher recurrence rate (15.2% vs 5.3%; P = 0.137), development of new metastases (27.3% vs 7.0%; P = 0.013) and higher mortality rate (15.2% vs 1.8%; P = 0.024). In the group of increased methylation level, patients had lower overall survival (P = 0.014) (Fig. 5) during the follow-up period.

**Table 4:** Results of mSEPT9 and CEA in colorectal cancer patients after surgery.

Cases		Time Point ^#^	Positive mSEPT9	Negative mSEPT9	Positive CEA	Negative CEA	P Value ^*^
All patients after surgery	6 M		38 (44.7%)	47 (55.3%)	22 (28.2%)	56 (71.8%)	0.035
	12 M		27 (51.9%)	25 (48.1%)	23 (31.9%)	49 (68.1%)	0.028
	18 M		15 (46.9%)	17 (53.1%)	18 (32.1%)	38 (67.9%)	0.180
	24 M		7 (63.6%)	4 (36.4%)	14 (34.1%)	27 (65.9%)	0.095
Patients without recurrence	6 M		34 (44.7%)	42 (55.3%)	19 (27.1%)	51 (72.9%)	0.038
	12 M		25 (51.0%)	24 (49.0%)	20 (30.3%)	46 (69.7%)	0.033
	18 M		11 (40.7%)	16 (59.3%)	15 (28.3%)	38 (71.7%)	0.316
	24 M		7 (63.6%)	4 (36.4%)	14 (34.1%)	27 (65.9%)	0.095
Patients with recurrence	6 M		4 (50.0%)	4 (50.0%)	3 (37.5%)	5 (62.5%)	1.0
	12 M		1 (50.0%)	1 (50.0%)	2 (40.0%)	3 (60.0%)	1.0
	18 M		4 (80.0%)	1 (20.0%)	3 (100.0%)	0 (0.0%)	1.0
	24 M		0	0	0	0	NA

mSEPT9, methylated septin 9; CEA, carcinoembryonic antigen; M, month.

^#^Time point was arranged as a time period, for example, 6 M concluded samples tested at 3 M and 6 M.

^*^P Value is the difference of negative percentages between mSEPT9 and CEA.

## Discussion

The overall sensitivity of mSEPT9 for the diagnosis of CRC was found to vary from 48.2% to 95.6% and the specificity was 79.1 to 99.1% in previous reports^17–21^. The second generation of mSEPT9, however, was found to have a higher sensitivity (71.1 to 95.6%) and similar specificity^21^. In the largest multi-center prospective study that evaluated the role of mSEPT9 in screening asymptomatic subjects (PRESEPT study)^17^, the overall sensitivity of the first-generation assay was 48.2%, which increased from stage I (35%) to stage IV (77.4%) cancers. As yet, the study used duplicate rather than triplicate PCR reactions, which may lower the sensitivity. To date, there is no consensus on the diagnostic algorithm of mSEPT9 PCR assay, studies that defined ‘one out of three positive reactions’ as positive usually have higher sensitivities for cancer. The FDA also approved the commercially available SEPT9 assay based on the data from the reanalyzed PRESEPT trial that used the ‘one out of three positive PCR reactions’ as positive sample^11,17^, which was also adopted in this study.

Using the latest second-generation mSEPT9 assay, we found a significantly higher sensitivity of mSEPT9 than CEA for the diagnosis of CRC patients (73.2% vs 48.2%; P < 0.001), especially for patients with stage II and III cancer (both P < 0.01), but a slightly lower specificity for the former (P > 0.05). Toth *et al*. reported similar results, with respective sensitivities of 95.6% (88/92) and 51.8%(14/27), and specificities of 84.8% and 85.2% for mSEPT9 and CEA^20^. In another recent study, mSEPT9 was also shown to have higher diagnostic value than CEA for both sensitivity (61.8% vs 35.0%) and specificity (89.6% vs 62.6%)^22^. The sensitivity of plasma mSEPT9 for detecting CRC was highly tumor stage-dependent. As shown in Fig. 2, the sensitivity increased progressively from stage I to IV. This rising positive rate with higher tumor stage has also been found in other studies^11–13^. It is however interesting to note in our study that the sensitivity of mSEPT9 in detecting proximal and distal cancers varied less than CEA, which would be an advantage for mSEPT9. This point was also confirmed in a previous study, which showed similar sensitivity of mSEPT9 for both right- and left-sided CRC (94% and 96%, respectively)^20^. In our study, both mSEPT9 and CEA were not sensitive enough in identifying patients with adenomas, including advanced and non-advanced adenoma, with all positive rates of less than 27.6%. In contrast, using the same Epi proColon 2.0 assay, Song *et al*. reported a high positive rate of mSEPT9 in villous adenoma and adenoma with high-grade dysplasia (83.3% and 62.5%, respectively) though the positive rate for all adenomas was much lower (31.8%)^13^. In this study, we also found that when taking the combined result of mSEPT9 and CEA, there was a significantly higher sensitivity and accuracy than single CEA (both P < 0.01) or mSEPT9 (P = 0.039 and 0.532) for diagnosing CRC.

More importantly, we prospectively compared the performance of mSEPT9 with CEA as a blood-based biomarker in the post-operative surveillance of CRC patients. The overall negative rates of mSEPT9 are lower than CEA from 6-months post-operatively, especially in patients with non-advanced cancer and those with no tumor recurrence. Among cancer patients with a positive mSEPT9 or CEA before operation, only about half of them (46.8% and 46.7%, respectively) turned negative within 6-months after surgery, suggesting both markers may not be sensitive enough for monitoring early treatment response. On the other hand, both mSEPT9 and CEA levels tended to be higher among patients with poor prognostic factors including tumor recurrence, detection of new metastasis and death. We observed a subgroup of cancer patients who had increased in quantitative levels of mSEPT9 after operation, which were associated with significantly higher rates of new metastases and mortality (both P < 0.05). Tham *et al*. used an in-house quantitative mSEPT9 assay and found that increased methylation levels of SEPT9 one year after surgery was associated with CRC recurrence, while the change at 6-month after surgery did not have any correlation^18^. In addition, Fu *et al*. found that the CRC patients with continued positive mSEPT9 after operation had closer correlation with recurrences or metastases within 12-months than those whose positive mSEPT9 turned negative^23^.

Our study had some limitations. First, our sample size for the study may not be large enough for the stratified analysis of cancer patients with different stages. As we only selected patients who underwent curative resection, the number of patients with advanced cancer staging and hence, adverse clinical outcomes such as death or clinical recurrence was relatively low during the initial follow up period, which make it difficult to conclude the roles of these biomarkers in detecting recurrence. More studies with larger sample sizes would be needed to increase precision of the estimates. Although the increased methylation levels of mSEPT9 have been shown in this study to be associated with higher mortality and new metastasis, the low rates of conversion from positive to negative in mSEPT9 during early post-operative period, may imply that mSEPT9 is not sensitive enough during this period. Due to the qualitative nature of the commercially available mSEPT9, it may still be inferior to CEA which is a quantitative assay on monitoring of treatment response. There are, however, increasing doubts that CEA may not be the best surveillance biomarker for CRC recurrence^24,25^. Shinkins *et al*. showed that CEA was not a satisfactory predictor for CRC recurrence regardless of the cut-off threshold in a total of 582 CRC patients after curative resection^24^. The recommended threshold of 5 ug/L would miss half the recurrences (53.8%; 95% CI: 44.2–64.4%) while the threshold of 2.5 ug/L would get very high rate of false alarms (84.2%; 924/1097 referred).

In conclusion, we compared the performance of mSEPT9 with CEA both for the diagnosis of CRC and post-operative monitoring of CRC patients. We found that the second generation of commercially available plasma mSEPT9 assay was more sensitive than CEA for the diagnosis of CRC patients, which parallels with the increase in tumor staging. Combined mSEPT9 and CEA would result in even higher sensitivity than either CEA or mSEPT9 alone. Both mSEPT9 and CEA values showed increasing trend among patients with poor prognosis after operation. Moreover, increased methylation level of mSEPT9 after surgical resection may be able to identify a subgroup of patients with adverse outcomes. Further studies may be needed to identify a more sensitive way of detecting minor changes in circulating mSEPT9 in blood for the detection of early CRC recurrence after surgery.

## Methods

### Participants

This was a prospective study conducted in the Queen Mary Hospital of Hong Kong, a university teaching hospital. For determination of the diagnostic performance of mSEPT9 in subjects with different colorectal neoplasm ranging from non-advanced adenoma, advanced adenoma to carcinoma, we prospectively enrolled patients undergoing colonoscopy in the Endoscopy Center of the hospital. These included symptomatic patients who were referred for colonoscopy due to bowel symptoms or diagnostic work up of iron deficiency anemia as well as patients undergoing screening colonoscopy. We excluded patients with previous bowel resection, familial colorectal cancer syndrome, inflammatory bowel disease or diagnosis of any other malignancy in the past. After obtaining informed consent, venous blood was collected from patients prior to the colonoscopy. Plasma was separated by centrifugation and stored at −20 °C till further analysis. During colonoscopy, all polyps were removed for histological examination and lesions suspicious for colorectal cancer were biopsied. All histological samples were reviewed by experienced pathologists in the hospital. Patients were then classified into four groups according to the most advanced lesions found on colonoscopy as (1) adenocarcinoma, (2) advanced adenoma, (3) non-advanced adenoma, and (4) normal colonoscopy without any polyp or adenoma. Advanced adenoma was defined as a lesion with a diameter of 10 mm or above, with villous histology or the presence of high-grade dysplasia^26^.

To determine the role of mSEPT9 in the post-operative monitoring of CRC patients, we prospectively recruited patients who were newly diagnosed to have adenocarcinoma of colon or rectum and were scheduled for curative resection in our hospital. All patients had baseline blood samples taken immediately before surgery and then at three-monthly intervals for up to 24-month. The baseline demographic and tumor staging were retrieved for these patients. Tumor staging was classified as stipulated by the seventh edition of the American Joint Committee on Cancer TNM Classification^27^. All patients were then prospectively followed up in our colorectal clinic after operation. The charts were reviewed and the outcome of these patients were further verified by the centralized electronic patients’ record (ePR) of the Hospital Authority of Hong Kong which is a territory-wide health database that capture all clinical information of patients including deaths. Clinical relapse or recurrence was determined clinically by history, physical examination and relevant investigation findings. Imaging techniques including CT scan or PET-CT were arranged when clinically indicated to confirm the presence of distant or regional recurrence. Surveillance colonoscopy after surgical resection was performed in all patients according to current recommendation^28^.

Informed consent was obtained from all patients and the study protocol was approved by the Institutional Review Board of the Hospital Authority-Hong Kong West Cluster and University of Hong Kong (UW 12-489).

### Detection of methylated SEPT9 and CEA

All plasma samples were coded with a unique sample collection number without patient identifier, final diagnosis or timing of blood sampling to ensure adequate blinding of laboratory staff involved in the testing of mSEPT9. mSEPT9 was determined by a commercially available assay (Epi proColon 2.0; Epigenomics AG, Berlin, Germany) in an independent laboratory (DiagCor, Hong Kong) authorized by the manufacturer. All samples were analyzed without patient information by real time PCR in triplicate and the mSEPT9 assay was considered positive when at least one of the three PCR reactions was positive^17^. Positive and negative controls were included in all PCR amplifications. Actin was used as reference, the results of SEPT9 methylation level was calculated using method similar to the 2^(−∆∆CT)^ ^29^ with normalization to Actin.

CEA levels were determined by enzyme linked immunoassay in the Clinical Immunology Laboratory of our hospital. Abnormal or positive CEA was defined as a value above 3 ng/ml as recommended by the laboratory. Levels lower than this were considered negative in all analyses.

### Statistical analysis

The sensitivity and specificity of mSEPT9 and CEA was computed with the 95% confidence intervals (CI). Chi-squared or Fisher Exact test was used to compare categorical data. All statistical analyses were two-sided, and a statistically significant difference was established when P < 0.05. All analysis was performed by the IBM SPSS Statistics software (version 21; IBM, USA) and GraphPad Prism (version 7; GraphPad Software, San Diego, CA).

### Ethics approval and consent to participate

Informed consent was obtained from all patients and the study protocol was approved by the Institutional Review Board of the Hospital Authority Hong Kong West Cluster and University of Hong Kong (UW 12-489). In addition, all research was performed in accordance with the Declaration of Helsinki – Ethical Principles for Medical Research involving Human Subjects.

## Supplementary information

Supplentary Information
