# Supplementary material for: Methylated Septin 9 and Carcinoembryonic Antigen for Serological Diagnosis and Monitoring of Patients with Colorectal Cancer After Surgery
Source: Sci Rep. 2019 Jul 17;9:10326. doi: 10.1038/s41598-019-46876-4 (PMC6637112; doi:10.1038/s41598-019-46876-4)
Supplement: Supplementary file 1 — Supplentary Information [file 41598_2019_46876_MOESM1_ESM.pdf]

METHYLATED SEPTIN 9 AND CARCINOEMBRYONIC ANTIGEN FOR SEROLOGICAL DIAGNOSIS AND MONITORING OF PATIENTS WITH COLORECTAL CANCER AFTER SURGERY

Zhi Yao Ma[1], Wai Lun Law[2], Enders Kai On Ng[3], Cherry Sze Yan Chan[1], Kam Shing Lau[1], Yuen Yee Cheng[4], Vivian Yvonne Shin[2], Ava Kwong[2], Wai K. Leung[1]

[1]Department of Medicine, University of Hong Kong, Queen Mary Hospital, Hong Kong
[2]Department of Surgery, University of Hong Kong, Queen Mary Hospital, Hong Kong
[3]DiagCor Bioscience, Kowloon Bay, Hong Kong
[4]Asbestos Diseases Research Institute, Sydney Medical School, The University of Sydney

Supplementary Table S1. CT values of 61 colorectal cancer patients at different time points before and after surgery

No. #	Recurrence	New Metastases	Death	Poor Prognosis *	Pre-operation: CT Value 1st Actin ##	Pre-operation: CT Value 1st SEPT9 ##	Pre-operation: CT Value 2nd Actin	Pre-operation: CT Value 2nd SEPT9	Pre-operation: CT Value 3rd Actin	Pre-operation: CT Value 3rd SEPT9	Pre-operation: Mean Value of SEPT9 Methylation Level **	Follow-up Time point 1: Months Post-operation	Follow-up Time point 1: CT Value 1st Actin	Follow-up Time point 1: CT Value 1st SEPT9	Follow-up Time point 1: CT Value 2nd Actin	Follow-up Time point 1: CT Value 2nd SEPT9	Follow-up Time point 1: CT Value 3rd Actin	Follow-up Time point 1: CT Value 3rd SEPT9	Follow-up Time point 1: Mean Value of SEPT9 Methylation Level	Follow-up Time point 2: Months Post-operation	Follow-up Time point 2: CT Value 1st Actin	Follow-up Time point 2: CT Value 1st SEPT9	Follow-up Time point 2: CT Value 2nd Actin	Follow-up Time point 2: CT Value 2nd SEPT9	Follow-up Time point 2: CT Value 3rd Actin	Follow-up Time point 2: CT Value 3rd SEPT9	Follow-up Time point 2: Mean Value of SEPT9 Methylation Level
1	No	Yes	No	Yes	26.16	37.2	26.16	45 ##	25.88	34.93	0.009	3M	24.63	45	24.8	40.45	24.88	45	0.000	18M	25.05	32.46	24.96	31.83	25.17	32.21	0.091
2	No	No	No	No	26.51	45	26.57	36.55	27.03	45	0.004	3M	26.84	37.08	26.61	45	26.57	45	0.004	24M	28.26	45	28.31	37.08	28.47	45	0.009
3	Yes	No	No	Yes	25.8	45	25.66	36.65	25.71	45	0.002	6M	26.73	45	26.49	35.83	26.91	45	0.006	18M	26.02	40.44	26.17	45	26.23	45	0.000
4	No	No	No	No	30.36	45	29.89	45	29.52	45	0.000	6M	27.79	45	27.53	45	27.32	45	0.000	12M	26.5	45	27.02	45	27.19	45	0.000
5	No	No	No	No	30.65	45	30.21	45	30.36	45	0.001	6M	26.42	45	26.23	45	26.3	45	0.000	12M	27.28	45	26.81	45	26.85	45	0.000
6	No	No	No	No	28.38	45	28.51	45	28.41	36.96	0.009	6M	27.95	45	27.99	45	28.16	45	0.000	12M	27.11	45	27.16	45	27.15	45	0.000
7	No	No	No	No	29.01	45	29.07	45	28.68	35.9	0.022	3M	27.37	45	27.38	45	27.4	45	0.000	24M	27.27	45	27.24	45	27.22	45	0.000
8	Yes	Yes	Yes	Yes	27.73	34.51	27.12	34.73	27.55	35.12	0.086	6M	32.19	45	32.18	45	32.12	45	0.002	15M	23.35	24.33	23.52	24.46	23.44	24.37	6.547
9	No	No	No	No	26.7	35.78	26.4	37.06	26.53	36.19	0.016	3M	27.2	45	27.35	45	26.84	45	0.000	24M	27.17	45	27.21	36.93	27.21	45	0.005
10	No	No	No	No	25.02	36.94	25.25	45	24.92	45	0.001	3M	26.7	45	26.61	45	26.61	45	0.000	21M	27.36	43.69	27.46	36.4	27.58	45	0.008
11	No	No	No	No	30.35	45	30.2	45	30.17	45	0.000	6M	28.62	45	28.45	45	28.68	45	0.000	12M	27.04	37.44	27.44	36.21	Undetermined	45	0.014
12	No	No	No	No	28.93	45	28.73	45	28.85	45	0.000	6M	27.29	45	27.18	45	27.33	45	0.000	12M	22.7	45	22.88	35.88	22.73	35.63	0.001
13	Yes	Yes	Yes	Yes	26.91	33.69	26.58	33.74	26.66	33.67	0.102	3M	27.09	29.6	27.3	29.74	27.42	29.76	2.332	6M	23.05	23.57	23.53	23.75	23.79	24.08	9.899
14	No	No	No	No	27.36	45	27.39	37.06	26.89	45	0.005	6M	29.06	39.27	29.18	45	29.25	45	0.004	12M	27.85	45	28.02	45	28.31	45	0.000
15	No	No	No	No	25.77	45	25.34	35.71	25.55	35.1	0.007	3M	28.06	45	28	45	28.53	45	0.000	15M	33.82	45	32.97	45	32.76	45	0.004
16	No	Yes	No	Yes	28.09	35.26	28.31	45	28.1	36.92	0.044	6M	28.89	38.31	28.63	45	28.55	35.48	0.035	24M	27.45	45	27.45	45	27.06	45	0.000
17	No	Yes	Yes	Yes	28.36	39.2	27.83	45	27.98	45	0.003	3M	29.7	41.22	29.43	45	30.23	45	0.002	6M	27.63	35.43	27.56	34.31	27.68	35.53	0.076
18	No	No	No	No	29.58	45	29.79	37.19	29.55	45	0.024	6M	26.55	45	26.26	45	26.23	45	0.000	12M	27.05	45	26.77	45	26.96	45	0.000
19	No	No	No	No	29.08	33.07	28.93	32.53	29.39	32.71	1.000	6M	25.61	45	25.49	45	25.45	44.38	0.000	12M	26.36	45	26.42	45	26.38	45	0.000
20	No	No	No	No	30.16	45	30.69	36.87	30.88	45	0.056	6M	25.8	45	25.84	45	26.39	37.63	0.001	12M	26.25	45	26.82	45	26.94	40.87	0.000
21	No	No	No	No	28.56	45	28.56	37.02	28.88	45	0.011	6M	26.3	35.98	26.53	37.75	26.56	36.32	0.012	15M	25.41	45	25.64	45	25.49	37.9	0.001
22	No	No	No	No	26.8	45	27.04	45	27.42	45	0.000	3M	24.53	45	24.36	37.93	24.27	45	0.000	18M	26.9	45	26.97	45	26.95	45	0.000
23	No	No	No	No	30.02	45	29.94	45	30.11	45	0.000	6M	31.11	45	32.19	45	31.8	45	0.001	15M	26.65	45	26.59	45	26.43	45	0.000
24	No	No	No	No	25.71	33.34	25.55	33.02	25.67	33.26	0.067	9M	27.8	45	27.54	45	27.78	45	0.000	18M	28.44	45	28.51	45	28.46	45	0.000
25	No	Yes	Yes	Yes	27.79	45	27.6	45	27.65	45	0.000	3M	27.07	45	27.84	45	27.99	45	0.000	15M	26.23	36.57	26.34	35.48	26.38	45	0.011
26	No	No	Yes	Yes	26.6	45	26.6	36.51	27	36.08	0.011	6M	27.14	34.4	27.18	34.29	27.11	34.82	0.080	9M	27.26	33.28	27.6	32.46	27.22	33.15	0.276
27	No	No	No	No	25.57	45	25.71	45	25.86	36.98	0.001	12M	26.08	45	26.08	38.01	25.97	45	0.001	18M	26.78	45	28.17	45	27.88	45	0.000
28	No	No	No	No	26.44	35.66	26.5	36.23	26.41	35.28	0.021	1M	28.27	45	28.34	38.28	28.2	38.31	0.007	24M	25.3	45	25.3	37.92	25.33	45	0.001
29	No	No	No	No	28.46	38.27	28.79	42.09	28.56	41.93	0.007	12M	26.9	45	27.15	45	26.79	45	0.000	21M	26.26	45	26.23	45	26.17	45	0.001
30	No	No	No	No	29.93	45	30.47	45	30.13	45	0.000	6M	24.89	36.88	24.75	45	24.72	37.2	0.002	21M	24.49	37.01	24.51	45	24.51	41.65	0.001
31	No	Yes	No	Yes	28.55	41.85	29.28	37.34	29.16	43.09	0.016	9M	26.01	28.34	26.07	28.44	26.11	28.34	2.537	21M	24.83	36.2	24.97	36.12	24.87	37.23	0.004
32	No	No	No	No	30.1	45	30.24	36.78	30.43	45	0.044	6M	29.44	45	29.32	45	29.39	45	0.000	12M	27.5	45	27.62	36.96	27.34	45	0.006

33	No	No	No	No	26.87	36.24	27	36.63	27.48	36.96	0.018	3M	26.11	45	26.06	37.37	26.05	45	0.002	18M	25.67	45	25.66	45	25.88	45	0.000
34	No	No	No	No	29.56	33.95	29.08	34.52	29.45	35.7	0.388	3M	28.23	45	28.18	45	27.98	45	0.000	15M	28.96	45	28.88	45	29.32	45	0.000
35	Yes	Yes	No	Yes	28.22	36.03	27.93	35.84	28.11	36.83	0.048	6M	25.48	45	25.84	36.88	26.18	45	0.002	18M	22.54	24.49	22.59	24.49	22.61	24.48	3.360
36	Yes	Yes	No	Yes	28.9	45	28.51	45	28.89	36.32	0.019	6M	25.26	45	25.43	45	25.17	39.54	0.000	18M	28.2	36.08	28.22	36.2	28.2	37.18	0.045
37	No	No	No	No	27	38.87	27	37.89	27.29	36.45	0.009	6M	26.67	45	26.95	36.8	26.58	45	0.004	12M	25.91	45	25.67	45	25.86	45	0.000
38	No	No	No	No	28.38	45	28.67	37.85	28.8	45	0.007	6M	25.3	45	25.29	45	25.33	45	0.000	12M	25.45	45	25.68	45	25.79	45	0.000
39	No	Yes	No	Yes	30.45	45	30.44	45	30.47	45	0.001	6M	27.09	45	27.26	45	27.4	45	0.000	12M	23.97	45	23.98	37.87	23.95	36.88	0.001
40	No	No	No	No	26.31	27.48	26.15	27.52	26.24	27.51	5.303	6M	27.3	45	26.87	45	26.97	45	0.000	12M	26.91	45	26.52	45	26.55	45	0.000
41	No	No	No	No	27.5	45	27.58	35.65	27.53	36.56	0.021	6M	24.84	45	25.01	45	24.79	45	0.000	15M	26.13	37.26	25.78	45	26.07	36.85	0.004
42	No	No	No	No	27.65	45	27.79	38.56	27.71	45	0.002	3M	27.5	45	27.36	45	27.63	45	0.000	18M	25.33	45	25.3	45	25.3	45	0.000
43	No	No	No	No	28.68	45	28.99	45	28.88	45	0.000	3M	23.61	36.77	23.52	37.99	23.62	37.23	0.001	15M	27.18	45	26.85	45	26.87	45	0.000
44	No	Yes	No	Yes	29.3	37.36	29.35	41.88	29.34	38.96	0.025	6M	27.13	45	27.27	37.15	27.1	45	0.004	15M	26.99	40.01	27.1	45	27.13	45	0.001
45	No	No	No	No	26.54	31.57	26.72	31.75	26.87	31.78	0.396	9M	26.35	45	26.66	36.99	26.52	45	0.003	15M	26.76	33.71	26.83	34.29	26.88	33.03	0.113
46	No	No	No	No	24.46	45	24.74	45	24.69	34.85	0.003	9M	24.64	38.08	24.84	45	24.76	38.27	0.001	15M	25.72	45	25.88	45	25.64	45	0.000
47	No	No	No	No	28.32	33.17	28.58	33.5	28.47	34.08	0.385	3M	27.28	45	27.54	45	27.34	45	0.000	12M	22.58	36.21	22.63	28.92	22.63	37.5	0.052
48	No	No	No	No	28.63	38.68	28.9	45	28.54	45	0.005	6M	27.44	36.6	27.26	36	27.6	45	0.019	12M	27.63	45	27.92	36.64	28.04	45	0.010
49	No	No	No	No	27.74	45	27.53	45	27.79	45	0.000	3M	24.99	45	25.37	32.22	25.25	37.21	0.036	15M	27.31	45	27.4	45	27.29	45	0.000
50	No	No	No	No	26.95	45	27.38	45	27.53	45	0.000	6M	30.21	45	30.27	45	30.36	45	0.000	12M	25.6	45	25.56	45	25.85	45	0.000
51	No	No	No	No	27.24	36	27.23	34.84	27.22	34.27	0.058	6M	26.41	45	26.24	39.46	26.44	45	0.000	12M	26.79	45	26.7	45	26.86	45	0.000
52	No	No	No	No	25.82	35.92	25.85	39.51	25.74	36.48	0.007	3M	26.21	45	26.44	45	26.45	45	0.000	12M	26.84	45	26.9	35.18	26.6	45	0.013
53	No	No	No	No	27.52	38.51	27.56	37.28	27.31	45	0.007	6M	22.69	45	22.64	40.6	22.75	45	0.000	12M	25.19	45	25.2	45	25.23	45	0.000
54	Yes	Yes	No	Yes	25.89	35.24	25.97	35.41	25.92	35.3	0.019	1M	25.35	45	25.45	45	25.34	45	0.000	16M	26.45	45	26.59	45	26.4	45	0.000
55	No	No	No	No	27.63	45	27.69	45	27.73	45	0.000	9M	25.71	45	25.4	45	25.91	45	0.000	15M	28.01	45	28.82	36.96	28.39	45	0.014
56	No	No	No	No	27.34	45	27.15	45	26.95	45	0.000	6M	23.99	45	23.98	37.22	24.06	45	0.000	12M	27.08	36.92	27.14	45	27.15	45	0.006
57	No	No	No	No	25.34	30.45	25.1	30.64	24.97	30.3	0.323	6M	27.15	45	27.48	35.93	27.52	45	0.012	12M	24.84	45	24.9	45	24.97	45	0.000
58	No	No	No	No	26.57	32.4	26.75	31.09	26.63	31.83	0.383	3M	25.88	45	25.9	45	25.89	45	0.000	9M	26.12	45	26.38	45	26.27	36.87	0.002
59	No	No	No	No	25.44	39.17	25.28	33.92	25.49	33.91	0.020	6M	27.89	45	27.8	45	27.83	37.03	0.006	18M	25.87	45	26.13	45	25.75	45	0.000
60	Yes	No	No	Yes	26.85	45	26.58	45	26.73	45	0.000	3M	28.47	45	28.19	45	28.37	45	0.000	9M	27.44	45	27.9	45	27.85	45	0.000
61	No	No	No	No	27.48	45	27.33	42.44	27.37	45	0.000	6M	29.76	36.93	30.19	45	29.94	45	0.037	9M	23.42	45	23.56	36.78	23.69	45	0.000

[#] These 61 colorectal cancer patients were detected SEPT9 methylation completely before operation and 2 times after operation.

[*] Poor prognosis refers to any of the three bad prognostic factors, including recurrence, new metastases and death.

[##] CT criteria for ACTIN and SEPT9 were separately less than or equal to 32.1 and less than 45, the opposite value would show 'Undetermined', here all value of 45 represented undetermined SEPT9 CT value for calculating methylation level. Totally 61 patients had complete pre-operation and 2 time points follow-up samples, all these samples had calculated methylation levels.

[**] ACTIN was used as reference, the results of SEPT9 methylation level was calculated using method similar to the $2^{(-\Delta\Delta CT)}$ with normalization to ACTIN.

Supplementary Table S2. Carcinoembryonic antigen of colorectal cancer patients at different time points before and after surgery

No.	Recurrence	New Metastases	Death	Poor Prognosis *	CEA Pre-Operation	CEA (ng/ml) Post-operation									
						1W	1M	3M	6M	9M	12M	15M	18M	21M	24M
1	No	No	No	No	2	1.3	1.9	2.7			1.8		2		
2	No	Yes	No	Yes	4.2		3.8	12					39		66
3	No	No	No	No	2		0.6		0.9	0.6	0.7	0.9		2.4	
4	No	No	No	No	4.6	0.8	0.7	0.9	1.1						
5	No	No	No	No	0.8	1.2		3	2.1	1.8		1.8	1.8		1.6
6	No	No	No	No	3			3.6	3.5	2.3		2.1	2.3	3.2	
7	No	No	No	No	9.9	4.9		1.7	1.7	1.7		1.6	1.9	2.2	
8	No	No	Yes	Yes	4.5	5.5		8.8	9.3						
9	No	No	No	No	1.7		1.4	2	1.6	1.3	2.2	1.7			
10	No	No	No	No	6.4		2.4	4.4	4.8	3.1	3.2	3.1	3.7		4.8
11	No	No	No	No	1.4		0.7	1.3	1.2		1.4			1.2	
12	No	No	No	No	1.6	1.5		1.2	1.4	1.9	1.2				
13	No	No	No	No	1.1	0.5			2				0.7		
14	No	No	No	No	1.5	2	2	2.5	2.3	2.6	2.5		2.9	2.6	
15	No	No	Yes	Yes	58			12							
16	No	No	No	No	2.2	1.7		2.2	2.5	2.4	1.8		2.7	2.8	
17	No	No	No	No	7.3		1.8	1.8	1.8	2.5		2.4			2.6
18	No	No	No	No	1	0.8	1.2	1.5			1.1	1.2	1.2	1.1	1.1
19	No	No	No	No	1.7	1.1		2		2.3			2.1	2	
20	No	No	No	No	3.4	2.3		3.9	3.6				3.1		3.1
21	No	No	No	No	0.9		1.2	2	1.9	1.6	1.8	1.6			
22	No	No	No	No	3.9		1.5	1.4	1.1	0.9	1			0.7	
23	No	No	No	No	1.3	1.2	1.4	1.3	1.5	1.5				1.4	2
24	No	No	No	No	2.4		2	2.6	2.8	2.3	1.9	2.5			
25	No	No	No	No	7.7	2.7	2.9	3.2	2.9	2.1	3.2	3.9	2.9	3.2	
26	No	No	No	No	0.8	0.8		0.7	0.9	0.6	1.2	1.3			0.7
27	No	No	No	No	2.2	1.2	1.3		1.7	1.6	1.7			1.7	
28	No	Yes	No	Yes	1.3		0.7			1.1				1.6	4.2
29	No	No	No	No	2.1	1.4			2.9	1.6	1.9		3		2
30	No	No	No	No	2.4	1.6		2.2			3.5				2.8
31	Yes	No	No	Yes	2.2	2.1	1.3		2	2.3			4.4		
32	No	No	No	No	3.1		2.5		3.5	3.7	3	2.6	2.9		
33	No	No	No	No	6.2	4.1	4.2	6.5	4.6	3.4	5	5.4	5.3	6.9	6.2
34	No	No	No	No	2.2	1.1		2.2	2.5					1.4	1.8

35	Yes	Yes	Yes	Yes	19	7	6.2		9.7	7.9		5.3			
36	No	No	No	No	12	2.3		1.6		1.7		1.4		2	1.6
37	No	No	No	No	3.2		4.6	6.2	7.1					7.6	7.5
38	No	No	No	No	2.2		1.1	1.3	1.2	1.8	1.7				
39	Yes	Yes	Yes	Yes	36	7.6		116							
40	No	No	No	No	6.1			4.5	4.4		6.4		4.9	4.9	
41	No	No	No	No	5.4	2.4		1.4	2	2.1		2.6	2.7		3
42	No	No	No	No	20	6.8	1.8	1.3	1.8			1.4		1.7	
43	No	Yes	No	Yes	5.1		1.1	1.3	0.9	1					
44	No	Yes	Yes	Yes	9.2	1.3		1.8	1.3						
45	No	No	No	No	4.4		2.6		2.8		2.6				
46	No	No	No	No	7.4	5.8		6.1	4.9		4.4	5.3			3.5
47	No	No	No	No	2.4	1.6		2.6	2.5	2.7	2.2				2.5
48	No	No	No	No	2.2	0.8		1	1			1.4			
49	No	No	No	No	2.5			2.4			2.2		2.1		
50	No	No	No	No	1			1.1	1.1			0.9			
51	No	Yes	Yes	Yes	4		3.3	2.9				8.3			
52	No	No	Yes	Yes	6.8		3.4	6	27	40					
53	No	No	No	No	3.8	1.7					2.4		2.7		2.4
54	No	No	No	No	1.6									1.5	
55	No	No	No	No	1	0.7	0.7	0.7			1	1		1.3	
56	No	No	No	No	6.1	1.5	0.9	2.2	1.6	1.1			1.1	1.6	1.4
57	No	Yes	No	Yes	10		12			17	4.3			4.1	30
58	No	No	No	No	2.2				1		1		1.1		
59	No	No	No	No	4.5	4.2		4.4		5.3	6		4.7		5.3
60	Yes	Yes	No	Yes	8.8		2.1	17	2		3.1		143		
61	Yes	Yes	No	Yes	2.9	4.3			2.8	2.9					
62	No	No	No	No	1.5		1.4	1.9	2.4	2.2	2				
63	No	Yes	No	Yes	6.4		2.4		9.5		4.7				
64	No	No	No	No	4.1		4.8		1.9	2.1	3		2.1		
65	No	No	No	No	6		7.1		3	3		7.6	9.2		
66	No	No	No	No	29		6.7	6.4	9.5	11				5.2	
67	No	No	No	No	1.1	1		2.3		2	1.2	1.5	1.6		
68	No	Yes	No	Yes	1.7		2.5	2.2	2.5	3.5		3.1			
69	No	Yes	No	Yes	2.5		0.9		1.5			1.5			
70	No	No	No		36					4.5		5.1	6.5		
71	Yes	No	No	Yes	0.9		0.7	0.8		1.4					
72	No	No	No		2.2		0.9			0.9	1.5	1.3		1.7	

73	No	No	Yes	Yes	1.8									
74	No	No	No	No	2.7	2		2.5		2.4	2.9		1.3	
75	No	No	No	No	14		3.5		2.6	2.6	2.7			
76	No	No	No	No	3.3	2.8					4		5.2	
77	No	No	No	No	5.7	1.9			2.7		3	4.5		4.6
78	No	No	No	No	4.3		2.3	3.1	3.8	3.6	5.4		1.8	
79	No	No	No	No	2.7		1.2	2.2			2.3			
80	No	No	No	No	1.8		0.9		1.5	1.6	1.2		1.3	
81	Yes	Yes	No	Yes	6.7		1.2							
82	No	No	No	No	2.2					1.5		1.7	25	
83	No	No	No	No	24				6.2		6.2		2.2	
84	No	No	No	No	2.9				1.4	1.2	1.5		1.2	1.6
85	No	No	No	No	0.7		7.8	10		8.9				
86	No	No	No	No	3.2			1.1	1.2		1.2		1.1	
87	Yes	No	No	Yes	84			15	84					
88	No	No	No	No	1	0.7		0.8	0.9	0.9	1.1			
89	No	No	Yes	Yes	4				3.6					

* Poor prognosis refers to any of the three bad prognostic factors, including recurrence, new metastases and death.
